# Contemporary seasonal human coronaviruses display differences in cellular tropism compared to laboratory-adapted reference strains

**DOI:** 10.1128/jvi.00684-25

**Published:** 2025-08-27

**Authors:** Matthew J. Gartner, Monique L. Smith, Clyde Dapat, Yi Wen Liaw, Thomas Tran, Randy Suryadinata, Joseph Chen, Guizhi Sun, Rory A. Shepherd, George Taiaroa, Michael Roche, Wen Shi Lee, Philip Robinson, Jose M. Polo, Kanta Subbarao, Jessica A. Neil

**Affiliations:** 1 Department of Microbiology and Immunology, The University of Melbourne at the Peter Doherty Institute for Infection and Immunity, Melbourne, Victoria, Australia; 2 WHO Collaborating Centre for Reference and Research on Influenza, The Peter Doherty Institute for Infection and Immunity 534133 , Melbourne, Victoria, Australia; 3 Victorian Infectious Diseases Reference Laboratory at the Peter Doherty Institute for Infection and Immunity, Melbourne, Victoria, Australia; 4 Department of Respiratory Medicine, Royal Children’s Hospital, Melbourne, Victoria, Australia; 5 Infection and Immunity, Royal Children's Hospital, Murdoch Children's Research Institute 34361, Parkville, Victoria, Australia; 6 Department of Anatomy and Developmental Biology, Monash University 2541, Clayton, Victoria, Australia; 7 Development and Stem Cells Program, Monash Biomedicine Discovery Institute, Clayton, Victoria, Australia; 8 Australian Regenerative Medicine Institute, Monash University 2541, Clayton, Victoria, Australia; 9 Department of Infectious Diseases, The University of Melbourne at the Peter Doherty Institute for Infection and Immunity, Melbourne, Victoria, Australia; 10 Department of Paediatrics, The University of Melbourne at the Royal Children’s Hospital, Parkville, Australia; 11 Adelaide Centre for Epigenetics, University of Adelaide 1066, Adelaide, South Australia, Australia; 12 South Australian Immunogenomics Cancer Institute, University of Adelaide 1066, Adelaide, South Australia, Australia; 13 Department of Microbiology, Infectiology and Immunology, Centre de recherche du CHU de Québec, Laval University 4440, Quebec, Canada; Loyola University Chicago - Health Sciences Campus, Maywood, Illinois, USA

**Keywords:** seasonal coronavirus, air-liquid interface airway cultures, AT2 cells

## Abstract

Seasonal human coronaviruses (sHCoVs) cause 15%–30% of common colds. The reference strains used for research were isolated decades ago and have been passaged extensively, but contemporary sHCoVs have been challenging to study as they are notoriously difficult to grow in standard immortalized cell lines. Here, we addressed these issues by utilizing primary human nasal epithelial cells (HNECs) and immortalized human bronchial epithelial cells (BCi) differentiated at an air-liquid interface, as well as human embryonic stem cell-derived alveolar type II (AT2) cells to recover contemporary sHCoVs from human nasopharyngeal specimens. From 21 specimens, we recovered four HCoV-229e, three HCoV-NL63, and eight HCoV-OC43 viruses. All contemporary sHCoVs showed sequence differences from lab-adapted CoVs, particularly within the spike gene. Evidence of nucleotide changes in the receptor binding domains within HCoV-229e and detection of recombination for both HCoV-229e and HCoV-OC43 isolates was also observed. Importantly, we developed methods for the amplification of high-titer stocks of HCoV-NL63 and HCoV-229e that maintained sequence identity, and we established methods for the titration of contemporary sHCoV isolates. Comparison of lab-adapted and contemporary strains in immortalized cell lines and airway epithelial cells revealed differences in cell tropism, growth kinetics, and cytokine production between lab-adapted and contemporary sHCoV strains. These data confirm that contemporary sHCoVs differ from lab-adapted reference strains and, using the methods established here, should be used for the study of CoV biology and evaluation of medical countermeasures.

**IMPORTANCE:**

Zoonotic coronaviruses have caused significant public health emergencies. The occurrence of a similar spillover event in the future is likely, and efforts to further understand coronavirus biology should be a high priority. Several seasonal coronaviruses circulate within the human population. Efforts to study these viruses have been limited to reference strains isolated decades ago due to the difficulty in isolating clinical isolates. Here, we use human airway and alveolar epithelial cultures to recover contemporary isolates of human coronaviruses HCoV-NL63, HCoV-229e, and HCoV-OC43. We establish methods to make high-titer stocks and titrate HCoV-229e and HCoV-NL63 isolates. We show that contemporary isolates of HCoV-NL63 and HCoV-OC43 have a different tropism within the respiratory epithelium compared to lab-adapted strains. Although HCoV-229e clinical and lab-adapted strains similarly infect the respiratory epithelium, differences in host response and replication kinetics are observed. Using the methods developed here, future research should include contemporary isolates when studying coronavirus biology.

## INTRODUCTION

Coronaviruses (CoVs) are ubiquitous in a wide range of avian species and mammals, which can act as a reservoir for transmission to humans (1). Seven coronaviruses are known to infect humans via the respiratory tract, causing a spectrum of respiratory diseases. In the past two decades, three zoonotic CoVs, severe acute respiratory syndrome coronavirus (SARS-CoV), Middle East respiratory syndrome coronavirus (MERS-CoV), and SARS-CoV-2 have emerged from animals to cause significant public health emergencies (2, 3). In addition, four seasonal human CoVs (sHCoVs; HCoV-229e, HCoV-NL63, HCoV-OC43, and HCoV-HKU1) have been circulating in the human population for decades and are presumed to have zoonotic origins (4). sHCoVs generally cause self-limiting respiratory infections and account for 15%–30% of common cold cases (5). However, these viruses can lead to more severe respiratory disease in neonates, the elderly, and immunocompromised populations (6, 7).

While there has been a significant research effort to characterize and understand the recently emerging CoVs (SARS-CoV, MERS-CoV, and SARS-CoV-2), we have less knowledge of the biology and evolutionary dynamics of the sHCoVs. Like emerging CoVs, sHCoVs display genetic evolution over time predominantly within the Spike (S) protein, suggesting the emergence of viral variants with altered tropism or the ability to escape pre-existing antibody immunity may occur (8–10). Studies investigating host cell tropism, entry, replication, and assessment of medical countermeasures using sHCoVs are limited primarily to lab-adapted reference strains. These lab-adapted reference strains were isolated decades ago and have been extensively passaged in immortalized cell lines. Whether these lab-adapted strains accurately represent the contemporary strains circulating in the population is unclear. Isolation and propagation of contemporary sHCoVs from clinical specimens in immortalized cell lines has been challenging. A previous study published by Dijkman et al. in 2013 reported the use of human bronchial airway epithelial cells differentiated at an air-liquid interface (ALI) to isolate 10 contemporary sHCoV strains (11). This study demonstrated that one universal cell culture system could be used to isolate diverse sHCoV strains. However, the success rate of recovery of HCoV-NL63 isolates was lower than the other viruses, likely because the requirements for growth of each sHCoV differ. There was also limited characterization of sequence variability and replication of the contemporary isolates relative to lab-adapted strains.

Therefore, to overcome these issues and establish models to study contemporary sHCoVs, we have built on the methods of Dijkman et al. to use a combination of three models of the human respiratory tract: primary human nasal epithelial cells (HNECs) differentiated at an ALI, immortalized human bronchial epithelial cells (BCi) differentiated at an ALI, and embryonic stem cell-derived alveolar type II cells (AT2), to recover novel contemporary sHCoV isolates. We leveraged this suite of respiratory tract models (12) to isolate 15 contemporary sHCoVs, including HCoV-229e (*n* = 4), HCoV-NL63 (*n* = 3), and HCoV-OC43 (*n* = 8) from 21 nasopharyngeal swab specimens that were PCR-positive for sHCoV nucleic acids. We used next-generation sequencing to generate full-length genome sequences for each isolated sHCoV and performed phylogenetic analysis to understand genetic diversity among the isolates and published sequences. In addition, we established protocols for generating high-titer virus stocks using immortalized cell lines and compared replication kinetics of the contemporary and laboratory-adapted reference sHCoV strains in cell lines and complex airway models.

## RESULTS

### Isolation of contemporary sHCoVs using primary and stem cell-derived cell culture systems

**Fig 1:**
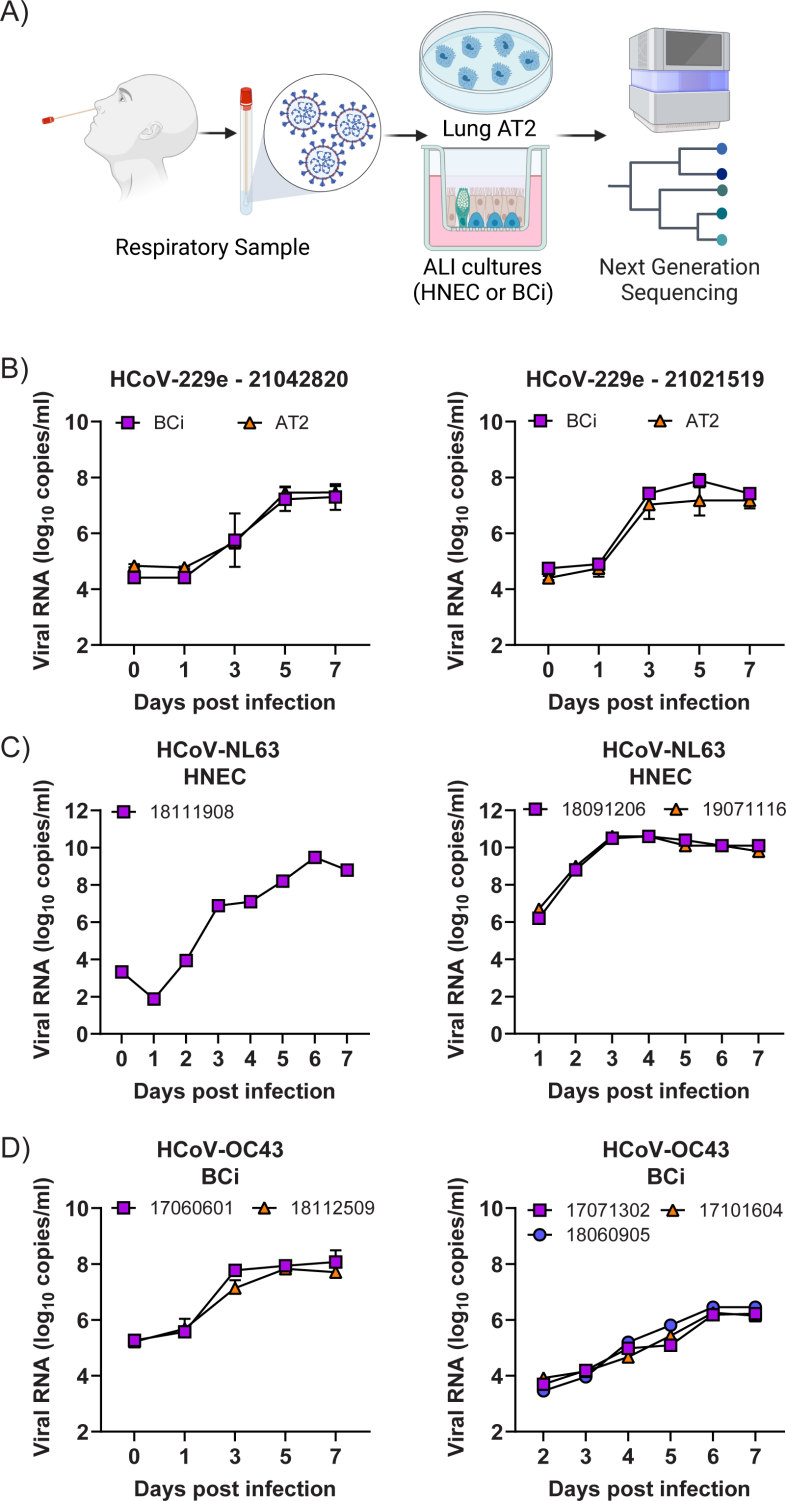
Isolation of contemporary sHCoVs. (**A**) Schematic of the procedure for recovering contemporary sHCoVs from human nasal swabs. Replication kinetics (Log_10_ genome copies/mL from 0 to 7 dpi) of recovered HCoV-229e isolates in immortalized BCi cells grown at an ALI and H9-derived AT2 cells (**B**), HCoV-NL63 isolates in primary HNECs grown at an ALI (**C**), and HCoV-OC43 isolates in immortalized BCi cells grown at an ALI (**D**). For (**B**), (**C**), and (**D**), either the mean ± SD from four unpooled replicates or the mean from four pooled replicates is shown. Each graph represents data generated from a single attempt to recover the virus using a nasal swab specimen.

We attempted to recover infectious virus from 21 nasopharyngeal specimens that were PCR+ for non-HCoV-HKU1 sHCoVs (HCoV-229e [*n* = 5], HCoV-OC43 or HCoV-NL63 [*n* = 16]) using human embryonic stem cell-derived AT2 cells, immortalized BCi cells differentiated at an ALI, or primary HNECs differentiated at an ALI (Fig. 1A). Receptors for each of these sHCoVs are shown in Table 1. We have previously shown that these cell types express ACE2 (12, 13). Comparison of nasal and bronchial epithelium indicates that higher expression of ACE2 is observed in the nasal epithelium (12, 14). Immunostaining of HNEC and BCi cultures showed similar human amino peptidase N (hAPN) expression between nasal and bronchial epithelial cells on the apical side of these cultures (Fig. S1A and B), similar to previous reports (11, 15). We also detected hAPN expression in Lung AT2 cultures (Fig. S1C). The expression of sialic acids is unknown. Nasopharyngeal specimens were collected over a period of 6 years, from 2017 to 2022. In brief, clinical specimens were diluted in phosphate-buffered saline (PBS) before incubation with the cells for 2 h at 33°C. Following the removal of inoculum, cells were cultured at 33°C for up to 7 days, and supernatant (apical wash for ALI cultures) was collected at various time points post-infection. For most experiments, supernatants from replicate wells were pooled to generate a virus stock and assessed for the presence of sHCoV nucleic acids by quantitative reverse transcription PCR (qRT-PCR). Two samples were lost due to contamination (likely due to the presence of yeast in the specimen). Of the remaining 19, four samples had no detectable levels of any virus at any time point when inoculated onto AT2 or BCi cells as determined by qRT-PCR (data not shown). Infectious sHCoVs were recovered from the remaining 15 specimens.

**TABLE 1:** Attachment factor, receptor expression, and cellular tropism of seasonal coronaviruses

Virus	Attachment factor	Receptor	Expression	Tropism	References
HCoV-229e	–^*a*^	APN	Airway epithelial cells (apical)	Ciliated and secretory epithelial cells	(11, 15, 16)
HCoV-NL63	Heparan sulfate proteoglycans	ACE2	Ciliated epithelial cells and alveolar type 2 cells	Ciliated epithelial cells and alveolar type 2 cells	(11–14, 17, 18)
HCoV-OC43	9-O-acetylated sialic acid	Unknown	Unknown	Ciliated epithelial cells	(11, 19)

^*a*^ –, none identified.

The five HCoV-229e+ specimens were inoculated on AT2 and/or BCi cells. As shown in Table 2, two samples were cultured in BCi cells only, one in AT2 cells only, and two in both cell types. Of the samples cultured in BCi cells only, one sample collected in 2018 did not yield virus. Infectious HCoV-229e was recovered from all other samples collected in 2021 and 2022. Samples 21042820, 21021519, and 22050721 grew robustly in AT2 cells, peaking at 5 to 7 days post-infection (dpi, Fig. 1B and data not shown). Cytopathic effect was observed from 3 dpi (data not shown). For samples 21021518, 21021519, and 21042820, similar growth kinetics were observed when the virus was recovered in BCi cells (Fig. 1B and data not shown). No obvious cytopathic effect (CPE) was observed in BCi cells that yielded virus (data not shown). Overall, 80% (4/5) of clinical samples containing HCoV-229e RNA yielded infectious virus from AT2 and BCi cells, with similar titers.

**TABLE 2:** Clinical specimens for isolation of seasonal coronaviruses

Sample no.	Specimen date (day/mo/yr)	CoV type	CT	Isolated in
No virus recovered				
17101603	16/10/2017	CoV	19	BCi
18103007^*a*^	30/10/2018	CoV	24	AT2
18171210^*a*^	12/07/2018	CoV	22	AT2
18041211	12/04/2018	HCoV-229e	23	BCi
19052913	29/05/2019	CoV	21	BCi
20060417	4/06/2020	CoV	24	AT2
HCoV-229e				
21021518	15/02/2021	HCoV-229e	23	BCi
21021519	15/02/2021	HCoV-229e	23	BCi + AT2
21042820	28/04/2021	HCoV-229e	21	BCi + AT2
22050721	7/05/2022	HCoV-229e	27	AT2
HCoV-NL63				
18091206	9/12/2018	CoV	25	HNEC + AT2
18111908	19/11/2018	CoV	20	HNEC + BCi
19071116	11/07/2019	CoV	22	HNEC + BCi
HCoV-OC43				
17060601	6/06/2017	CoV	19	BCi + AT2
17071302	13/7/2017	CoV	23	BCi
17101604	16/10/2017	CoV	20	BCi
18060905	9/06/2018	CoV	20	BCi
18112509	25/11/2018	CoV	19	BCi + AT2
18122712	27/12/2018	CoV	22	BCi
19052914	29/05/2019	CoV	20	BCi
19120715	7/12/2019	CoV	23	BCi

^*a*^ Lost due to contamination.

Three HCoV-229e negative specimens yielded HCoV-NL63: samples 18091206 and 18111908 collected in 2018 and 19071116 collected in 2019. While initial attempts to recover the virus from sample 18091206 in AT2 cells or 18111908 and 19071116 in BCi cells were unsuccessful, low levels of virus were detectable in either the inoculum or on 1 dpi in these cultures (Fig. S1B). Given that HCoV-NL63 primarily causes an upper respiratory tract infection and infects ACE2-expressing ciliated cells, we re-attempted virus isolation from these clinical samples using HNECs. All three viruses grew in HNECs, with virus peaking on 6 dpi for 18111908 and 3 dpi for 18091206 and 19071116 (Fig. 1C). The differential viral replication kinetics between 18111908 compared to 18091206 and 19071116 may be explained by the different HNEC donors used for virus isolation. In our hands, recovery of contemporary HCoV-NL63 viruses is most robust when using nasal epithelial cells compared to cells from lower in the respiratory tract.

The remaining eight clinical samples yielded HCoV-OC43. As shown in Table 2, they included three samples collected in 2017 (17060601, 17071302, and 17101604), three collected in 2018 (18060905, 18112509, and 18122712), and two collected in 2019 (19052914 and 19120715). Samples 17060601 and 18112509 were cultured in BCi and AT2 cells, while the remaining were only cultured in BCi cells. Attempts to recover samples 17060601 and 18112509 in AT2 cells were unsuccessful (Fig. S1D). All viruses were recovered in BCi cells, with virus peaking at days 3–6 dpi (Fig. 1D). This outcome was the opposite of that observed for the lab-adapted HCoV-OC43 strain (VR-1558), which showed robust replication in AT2 cells but no infection in BCi cells (Fig. S1E and F). This suggests that clinical HCoV-OC43 strains may show differences in host susceptibility and respiratory tract tropism compared to the lab-adapted reference strain.

### Genetic characterization of contemporary sHCoVs

**Fig 2:**
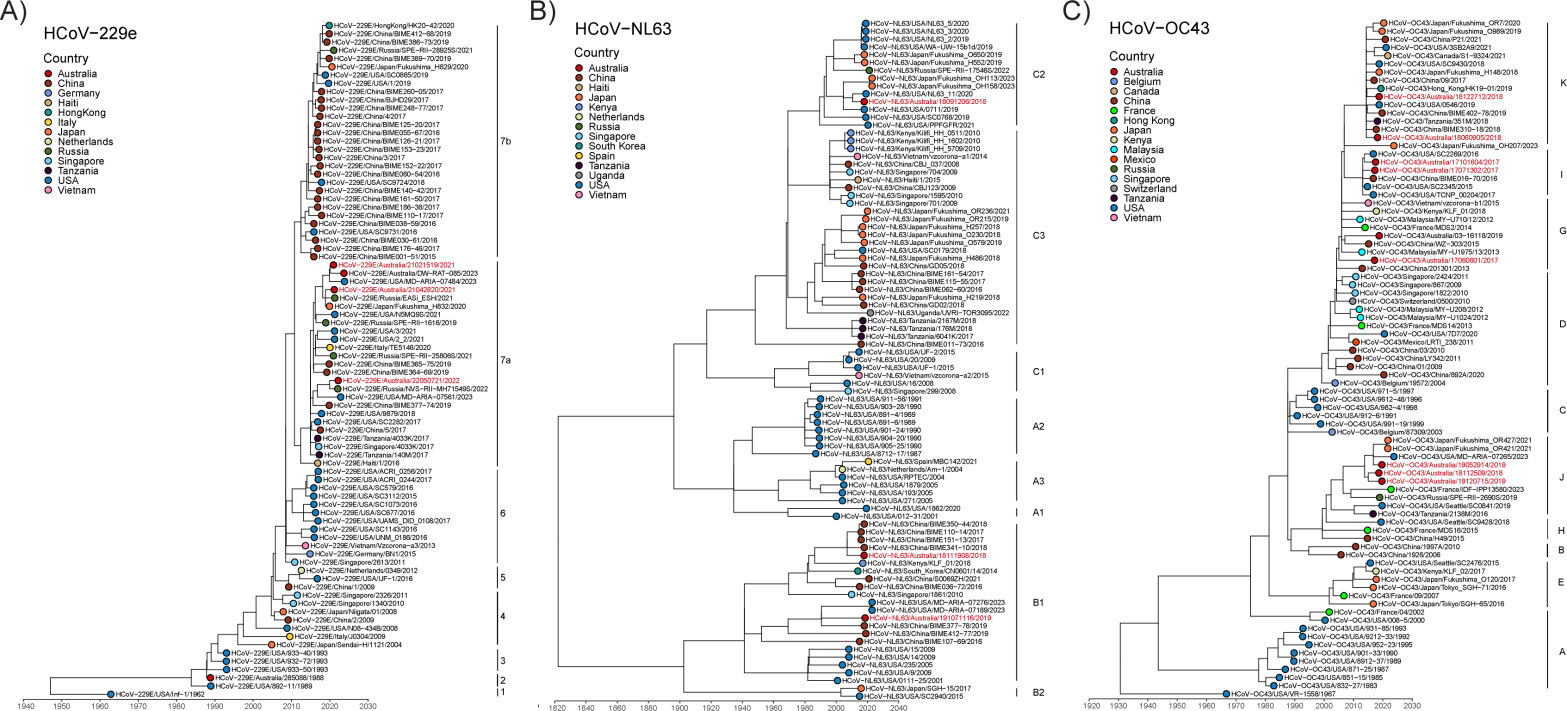
Genetic characterization of sHCoVs. Time-resolved maximum likelihood phylogenetic analysis of full genome nucleotide sequences of contemporary HCoV-229e (**A**), HCoV-NL63 (**B**), and HCoV-OC43 (**C**) isolates compared to publicly available sequences. Sequences from viruses isolated in this study are shown in red text. Colored symbols indicate different countries of origin.

To study the evolutionary relationships between the contemporary sHCoV isolates and other circulating CoVs, we generated full genome sequences for each contemporary sHCoV and built phylogenetic trees including publicly available genomes. For HCoV-229e, two isolates (21021519 and 21021518) had identical genome sequences and identical sample collection dates, so we removed 21021518 from further analysis. Consistent with previous studies, the HCoV-229e tree had a ladder-like shape with short terminal branches, and sequence divergence was proportional to virus isolation date, consistent with genetic drift (8–10, 20, 21). Our HCoV-229e isolates 21042820, 21021519, and 22050721 all fell within one clade (genotype 7a) containing sequences from 2017 to 2023 from diverse geographical locations, including Russia, Japan, Italy, and Tanzania (Fig. 2A), consistent with previous reports showing that HCoV-229e sequences cluster by date and not location (8, 21, 22). We noted two distinct clades of sequences with collection dates ranging from 2016 to 2023 (denoted genotypes 7a and 7b), suggesting the co-circulation of two different genotypes of HCoV-229e.

Analysis of HCoV-NL63 sequences showed our isolates grouped into two distinct genotypes; 18111908 and 19071116 clustered with sequences in genotype B1, while 18091206 grouped with sequences in genotype C2 (Fig. 2B). Similar to the HCoV-229e tree and consistent with other studies (9, 10, 20, 21, 23), the HCoV-OC43 tree showed sequences that diverged based on isolation date and not location, suggestive of genetic drift. Our HCoV-OC43 sequences showed the eight isolates grouped into four co-evolving lineages (Fig. 2C). Samples 18112509, 19052914, and 19120715 grouped with genotype J sequences, 17060601 grouped with genotype G, 17071302 and 17101604 grouped with genotype I, and 18060905 and 18122712 grouped with genotype K sequences (Fig. 2C).

**Fig 3:**
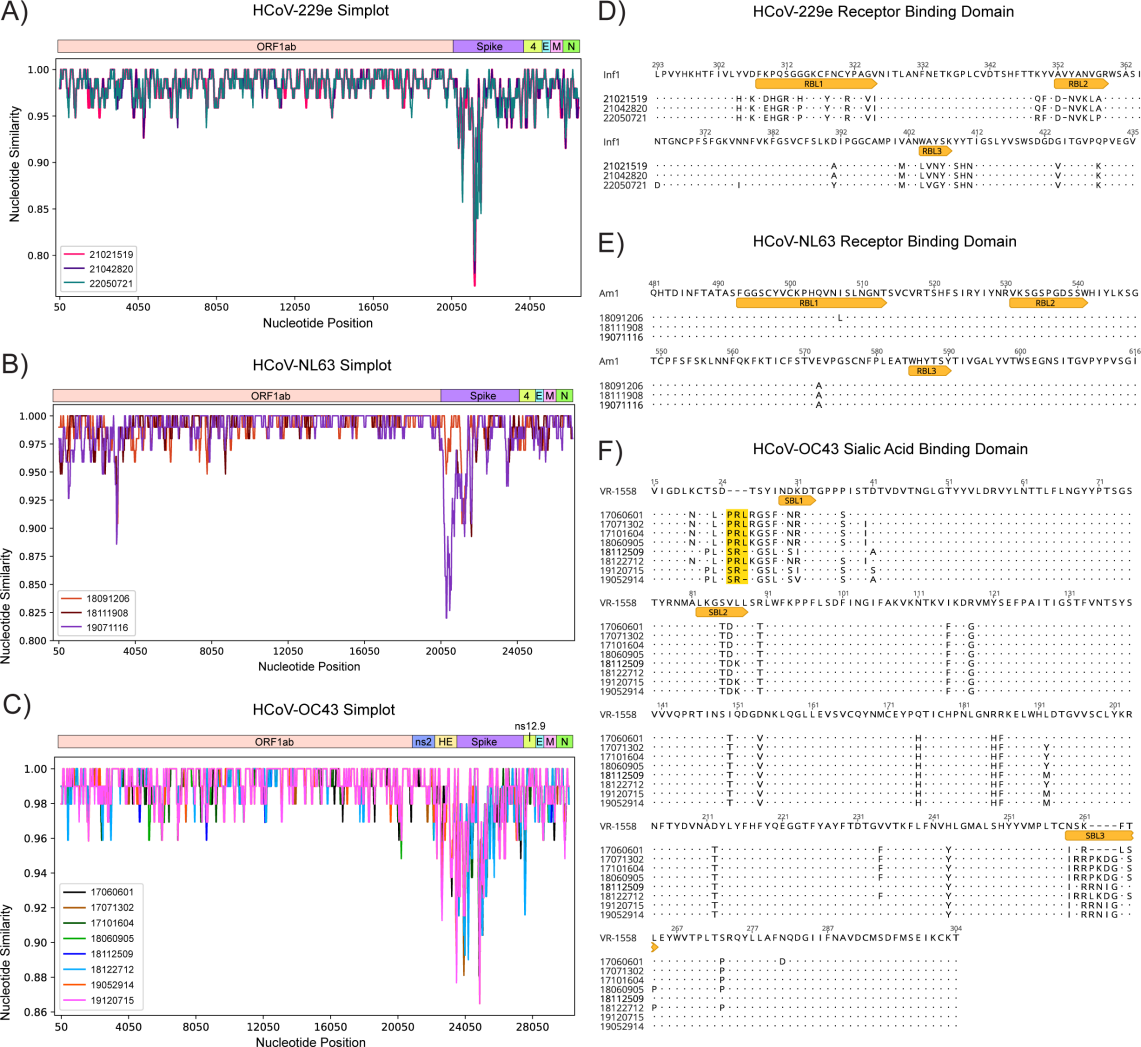
Sequence analysis of sHCoVs. Simplot analysis of full genome sequences of contemporary HCoV-229e (**A**), HCoV-NL63 (**B**), and HCoV-OC43 (**C**) isolates showing the percent nucleotide similarity across nucleotide positions compared to reference strains (HCoV-HCoV-229e/USA/Inf1/1962, HCoV-HCoV-NL63/NED/Amsterdam1/2004, and HCoV-HCoV-OC43/USA/VR-1558/1967, respectively). Analysis of the HCoV-229e receptor binding domain (**D**), HCoV-NL63 receptor binding domain (**E**), and HCoV-OC43 sialic acid binding domain (**F**) from contemporary isolates compared to the reference strain. RBL, receptor binding loop; SBL, sialic acid binding loop. Insertions upstream of SBL1 are highlighted.

We performed Simplot analysis and calculated nucleotide identity between each isolate compared to the respective reference strain to probe which viral genes demonstrated the greatest diversity. Simplot analysis of all three viruses demonstrated the highest genetic diversity within the S gene (Fig. 3A through C), with HCoV-229e isolates showing 95.51%–95.91% identity within S (Table 3), HCoV-NL63 showing 96.05%–98.16% identity within the S (Table 3) and HCoV-OC43 showing 95.50%–96.19% identity within the S compared to the respective lab-adapted reference strain (Table 4).

**TABLE 3:** Nucleotide identity of clinical isolates compared to HCoV-229e and HCoV-NL63 reference strains

Virus strain^*a*^	ORF1a	S	ORF4	E	M	N
HCoV-229e/USA/Inf-1/1962						
HCoV-229e/AUS/21021519/2021	98.51	95.80	97.12	98.72	98.23	97.09
HCoV-229e/AUS/21042820/2021	98.53	95.91	97.12	98.72	98.23	97.18
HCoV-229e/AUS/22050721/2022	98.47	95.51	97.12	99.15	98.23	97.52
HCoV-NL63/NLD/Am-1/2004						
HCoV-NL63/AUS/18091206/2018	99.12	98.16	99.12	98.72	97.95	99.29
HCoV-NL63/AUS/18111908/2018	99.16	96.27	99.26	99.15	95.87	99.56
HCoV-NL63/AUS/19071116/2019	99.00	96.05	99.41	98.29	98.53	99.12

^*a*^ AUS, Australia; NLD, the Netherlands.

**TABLE 4:** Nucleotide identity of clinical isolates compared to HCoV-OC43 reference strain VR-1558

Virus strain^*a*^	ORF1ab	Ns2	HE	S	Ns12.9	E	M	N
HCoV-OC43/USA/VR1558/1967								
HCoV-OC43/AUS/17060601/2017	99.25	98.92	97.49	95.78	99.39	99.62	98.27	98.52
HCoV-OC43/AUS/17071302/2017	99.23	98.92	97.33	95.50	99.09	99.62	98.27	98.52
HCoV-OC43/AUS/17101604/2017	99.19	99.16	97.25	95.55	99.09	99.62	98.27	98.59
HCoV-OC43/AUS/18060905/2018	99.21	99.16	97.18	95.47	99.09	99.62	98.27	98.66
HCoV-OC43/AUS/18112509/2018	99.20	99.16	97.02	96.19	98.79	96.93	98.56	98.29
HCoV-OC43/AUS/18122712/2019	99.20	99.16	97.10	95.50	99.09	99.23	98.27	98.74
HCoV-OC43/AUS/19052914/2019	99.20	99.16	97.10	96.16	98.79	96.93	98.56	98.44
HCoV-OC43/AUS/19120715/2019	99.18	99.16	96.94	96.13	98.79	96.93	98.56	98.37

^*a*^ AUS, Australia.

Given the diversity within S for HCoV-229e, HCoV-NL63, and HCoV-OC43, we generated alignments of predicted amino acid sequences for the S1 domain (Fig. S2A through C) and the receptor binding domain (RBD) of HCoV-229e and HCoV-NL63 (Fig. 3D and E) and the sialic acid binding domain (domain A) of HCoV-OC43 (Fig. 3F), comparing our isolates to the appropriate reference strain. Analysis of the RBD for HCoV-229e isolates showed several amino acid changes within the receptor binding loops (RBLs) 1, 2, and 3 compared to reference strain, Inf1 (Fig. 3D). The three RBLs are responsible for binding to hAPN for entry and undergo substantial genetic variation (8, 24). Outside the RBD, we found several amino acid changes within the N-terminal domain (NTD) compared to the reference strain (Fig. S2A), a region previously shown to be immunodominant in HCoV-229e S (25).

In contrast to HCoV-229e, we found minimal amino acid changes within the RBD of our HCoV-NL63 isolates compared to the reference strain Am-1 (Fig. 3E). Instead, we found some variation within the N-terminal Unique domain of S1 (Fig. S2B). Our HCoV-OC43 isolates showed substantial variation compared to the reference VR1558 strain near sialic acid binding loop 1 (SBL1), with three of the eight isolates having an SR insertion and the remaining five having a PRL insertion (Fig. 3F). In addition, all eight isolates showed variation in SBL2 and SBL3 compared to VR1558. The eight HCoV-OC43 isolates also showed substantial variation within domain B compared to VR1558 (Fig. S2C).

**Fig 4:**
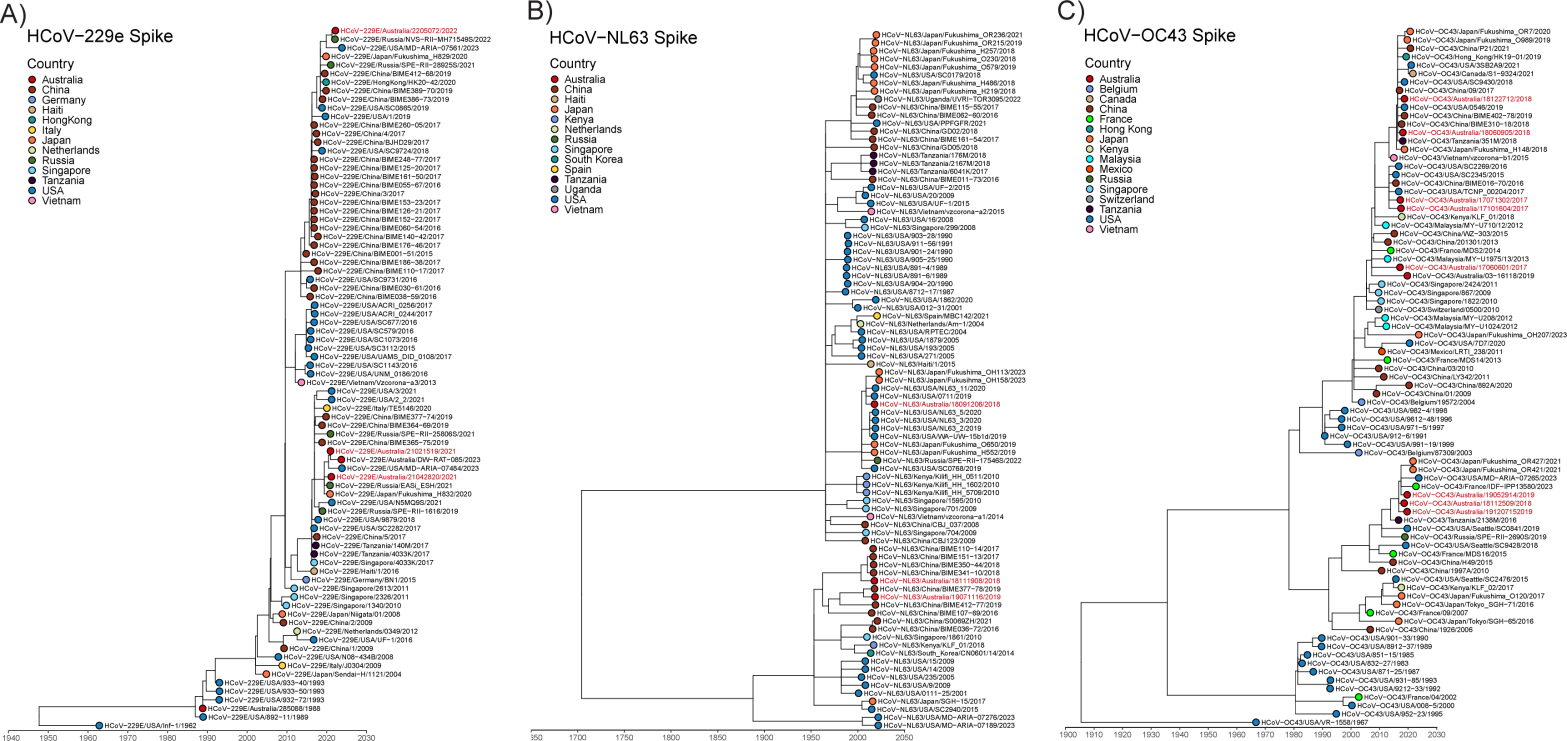
Alterations in sHCoV spike sequence. Time-resolved maximum likelihood phylogenetic analysis of spike nucleotide sequences of contemporary HCoV-229e (**A**), HCoV-NL63 (**B**), and HCoV-OC43 (**C**) isolates compared to publicly available sequences. Sequences from viruses isolated in this study are shown in red text. Colored symbols indicate different countries of origin.

We constructed phylogenetic trees using S gene sequences for HCoV-229e, HCoV-NL63, and HCoV-OC43 to understand the molecular evolution of the S gene over time (Fig. 4A through C). Similar to the trees based on the whole genome, we found two co-circulating genotypes of HCoV-229e S sequences over the same timeframe (2016–2023), indicating that two genotypes were circulating globally. Inconsistent with the full-length tree, 22050721 grouped with genotype 7b while 21021519 and 2106799 grouped with genotype 7a, suggesting 22050721 may be a recombinant. Recombination analysis using the sequences used to generate the phylogenetic tree predicted a recombination event in 22050721 between genotype 7a and 7b sequences with breakpoints occurring between ORF1ab and S (Fig. S3A).

Analysis of HCoV-NL63 S sequences showed 18111908, 19071116, and 18091206 grouped as they did in the full genome tree (Fig. 4B). Recombination analysis showed no evidence of recombination in our three HCoV-NL63 isolates (data not shown). Analysis of HCoV-OC43 S sequences showed all eight sequences grouped within the same genotype as the full genome tree (Fig. 4C). Recombination analysis of full genome HCoV-OC43 sequences identified a recombination event in 17101604 between a genotype G and K sequence with breakpoints between S and Nucleocapsid (N) (Fig. S3B).

### Growth of contemporary sHCoVs in immortalized cell lines

**Fig 5:**
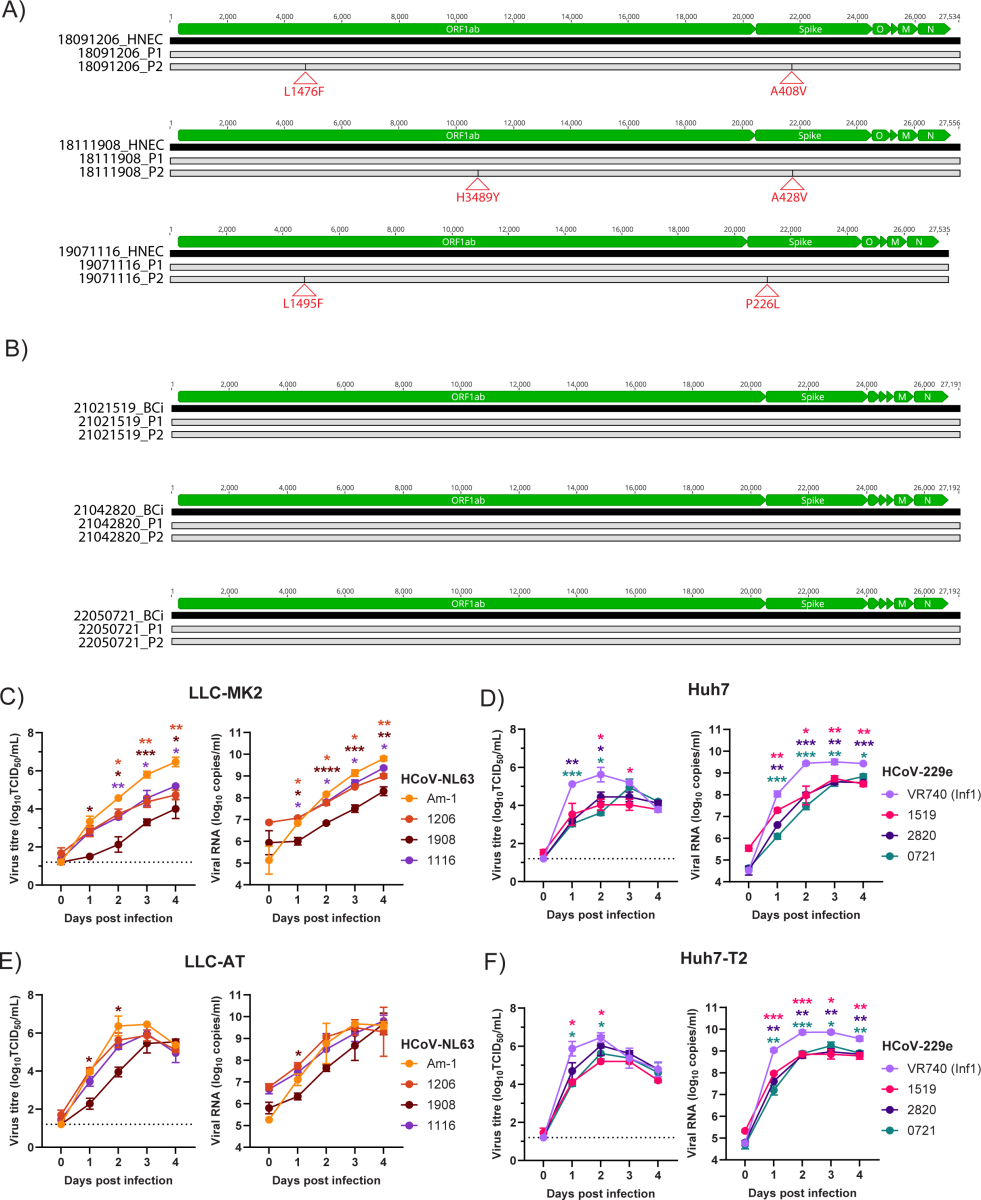
Comparison of virus growth in immortalized cell lines. Sequence alignments of (A) HCoV-NL63 and (B) HCoV-229e virus stocks isolated in HNEC (for HCoV-NL63) and BCi (for HCoV-229e) compared to passage 1 (P1) and passage 2 (P2) nucleotide sequences of the same virus produced in either LLC-AT (HCoV-NL63) or Huh7 (HCoV-229e) cells. Black lines indicate nucleotide changes, and red triangles indicate amino acid changes. Amino acid changes were only observed for the HCoV-NL63 P2 virus. Infectious virus titers and genome copies in LLC-MK2 (**C**) and LLC-AT (**D**) cells infected with lab-adapted (Am-1) and contemporary HCoV-NL63 isolates (18091206, 18111908, 19071116). Infectious virus titers and genome copies in Huh7 (**E**) and Huh7-T2 (**F**) cells infected with lab-adapted (VR740 [Inf1]) and contemporary HCoV-229e isolates (21021519, 21042820, 22050721). Data is a representative of at least two experimental repeats, each with three replicates. Mean ± SD is shown. Data were analyzed using a two-way analysis of variance (ANOVA) with a Dunnett’s post-test. *P < 0.05, **P < 0.01, ***P < 0.001, and ****P < 0.0001.

Downstream analysis of contemporary sHCoVs using supernatants from HNECs, BCi, and AT2 cells is difficult as these culture systems produce small volumes of supernatant. Therefore, we attempted to establish protocols to produce larger quantities of virus using immortalized cell lines. For HCoV-OC43, we attempted to grow the contemporary viruses in several cell lines, including MRC-5, Huh7, HCT-8, BHK-21, BCi.NS1.1, and MvLu1 cells, as well as Vero and A549 cells overexpressing human TMPRSS2. The lab-adapted HCoV-OC43 strain caused CPE and had detectable virus growth (by qRT-PCR or antigen detection) in all cell types. However, virus growth was not observed for any contemporary HCoV-OC43 isolates (data not shown). For HCoV-229e, we attempted to grow the isolates in Huh7 cells, as there are several publications that use this cell line to propagate the lab-adapted HCoV-229e virus (26–28). HCoV-229e isolates grew robustly and caused CPE in Huh7 cells. For HCoV-NL63, we attempted to grow the isolates in LLC-MK2 cells overexpressing ACE2 and TMPRSS2 (LLC-AT) as previously described (29, 30). Our HCoV-NL63 isolates grew robustly and caused CPE in LLC-AT cells. Given the propensity of CoVs to mutate on passage in immortalized cell lines, we performed whole genome sequence analysis of HCoV-229e and HCoV-NL63 isolates after two serial passages (P1 and P2) in Huh7 or LLC-AT cells, respectively. Comparison of HCoV-NL63 isolate consensus sequences after two passages in LLC-AT cells showed the accumulation of two amino acid changes per virus isolate, one each in ORF1ab and one each in S (18091206: L1476F in ORF1ab and A408V in S, 18111908: H3489Y in ORF1ab and A428V in S and 19071116: L1945F in ORF1ab and P226L in S, Fig. 5A). Notably, all amino acid changes that occurred within S were in the NTD. In contrast, comparison of HCoV-229e isolate consensus sequences after two passages in Huh7 cells revealed no genetic and amino acid changes (Fig. 5B).

To further explore the ability of contemporary sHCoVs to replicate in immortalized cell lines, we compared their replication kinetics (using P2 viruses) compared to the lab-adapted strains. All HCoV-NL63 viruses replicated in LLC-MK2 cells with titers peaking at 4 dpi (Fig. 5C). However, Am-1 replicated to significantly higher titers than the contemporary isolates, with 18111908 having the lowest titers over the 4 days (Fig. 5C). Genome copies over time were consistent with infectious virus levels (Fig. 5C). Overexpression of both human TMPRSS2 and ACE2 in the cell lines accelerated growth of all HCoV-NL63 viruses with infectious titers peaking at 2 dpi (Fig. 5D). While 18111908 still had slower growth compared to Am-1, the other two contemporary isolates grew to similar levels as Am-1 in both infectious virus titers and genome copies (Fig. 5D). Approximately 95%–100% CPE was observed in LLC-AT cells by 3 dpi for all HCoV-NL63 viruses. In comparison, CPE was only observed for Am-1 at 4 dpi in LLC-MK2 cells (data not shown). Overall, this suggests that the overexpression of ACE2 and TMPRSS2 significantly improves the replication of HCoV-NL63 isolates.

All HCoV-229e viruses replicated in Huh7 cells with titers peaking at 2 dpi (Fig. 5E and F). Overexpression of human TMPRSS2 increased the titers of HCoV-229e at 1 dpi compared to Huh7 cells, after which titers were similar between both cell types. Similar to the HCoV-NL63 isolates, all contemporary HCoV-229e isolates replicated to lower titers compared to the reference strain VR740 in Huh7 cells. Overexpression of TMPRSS2 modestly increased the replication of HCoV-229e isolates so that their replication was similar in kinetics to VR740. Overall, this suggests that TMPRSS2 expression has a modest effect on HCoV-229e infection, and Huh7 cells can be used to generate high-titer stocks of HCoV-229e.

### Comparison of infection with lab-adapted vs contemporary isolates in the respiratory tract

**Fig 6:**
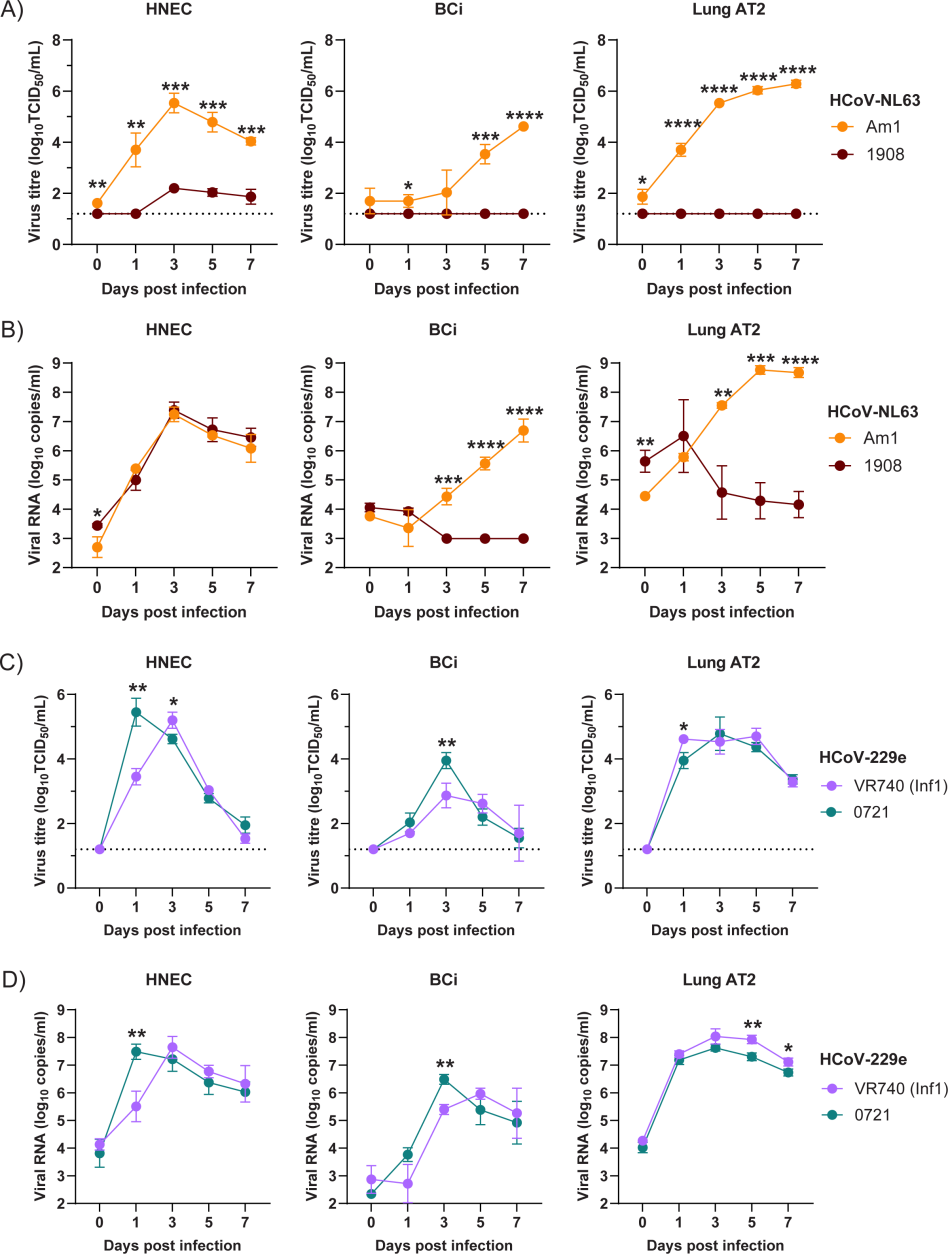
Comparison of virus growth across the human respiratory tract. Infectious virus titers (**A**) and genome copies (**B**) in HNECs, BCi, and AT2 cells infected with lab-adapted HCoV-NL63 (Am-1) or contemporary HCoV-NL63 18111908 (1908). Infectious virus titers (**C**) and genome copies (**D**) in HNECs, BCi, and AT2 cells infected with lab-adapted HCoV-229e (VR740-Inf1) or contemporary HCoV-229e 22050721 (0721). Data are representative of two experimental repeats, each with three replicates. Mean ± SD is shown. Data were analyzed using a student’s t test. *P < 0.05, **P < 0.01, ***P < 0.001, and ****P < 0.0001.

We compared replication kinetics of lab-adapted and contemporary HCoV-229e and HCoV-NL63 sHCoV strains across our human respiratory tract cell culture models. For HCoV-NL63, both Am-1 and isolate 18111908 replicated similarly in HNECs as determined by genome copies, but infectious titers were significantly lower for 18111908 compared to Am-1 over the 7 days (Fig. 6A and B). It is unclear whether this discrepancy is related to the presence of defective genomes or the ability to cause CPE in LLC-AT cells used in the infectivity assay. In contrast, only Am-1 replicated in BCi and AT2 cells (Fig. 6A and B). This is consistent with what we observed in isolating HCoV-NL63 from nasal swabs—contemporary HCoV-NL63 isolates preferentially infect the upper respiratory tract. In addition, it indicates a distinct difference in cell tropism between contemporary HCoV-NL63 and lab-adapted HCoV-NL63 viruses.

For HCoV-229e, both VR740 and 22050721 replicated in HNECs, BCi, and AT2 cells (Fig. 6C and D). Clinical isolate 22050721 had higher titers at 1 dpi in HNECs and 3 dpi in BCi compared to VR740. In contrast, VR740 had modestly higher infectious virus titers on 1 dpi and genome copies on 5 and 7 dpi compared to 22050721 in AT2 cells. Again, this is consistent with what we observed in isolating HCoV-229e from nasal swabs—contemporary HCoV-229e viruses were able to infect all parts of the respiratory tract. We found that 22050721 shows similar replication kinetics to lab-adapted HCoV-229e.

**Fig 7:**
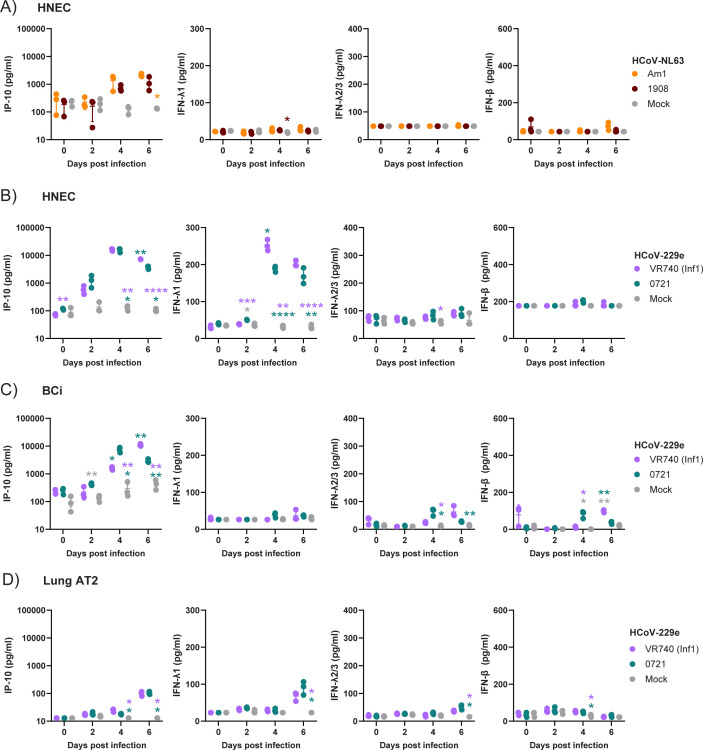
Comparison of the host response to sHCoVs. IP-10, IFN-λ1, IFN-λ2/3, and IFN-β1 levels in the supernatant of HNECs infected with HCoV-NL63 18111908 or Am-1 (**A**), HNECs infected with HCoV-229e VR740 or 22050721 (**B**), BCi infected with HCoV-229e VR740 or 22050721 (**C**), and AT2 cells infected with HCoV-229e VR740 or 22050721 (**D**). Data is a representative of two independent experiments. Individual replicates and mean ± SD are shown. Data were analyzed using a two-way ANOVA with Tukey’s post-test. *P < 0.05, **P < 0.01, and ***P < 0.001.

To further understand how infection with contemporary sHCoVs differs from lab-adapted strains, we measured cytokine responses (IP-10, IFN-λ1, IFN-λ2/3, and IFN-β). In HNECs that were able to support replication of both lab-adapted and contemporary HCoV-NL63, infection was associated with increased IP-10 secretion between 4 and 6 dpi compared to the mock control, reaching significance only for Am-1 at 6 dpi (Fig. 7A). No significant difference in the level of IP-10 secretion was observed between strains, and very little change in IFN-λ1, IFN-λ2/3, and IFN-β secretion was observed. Thus, HCoV-NL63 appears to induce minimal induction of IP-10 and type I/III interferons, with a similar response between strains.

In contrast to HCoV-NL63, HCoV-229e infection strongly induced the secretion of both IP-10 and IFN-λ1 in HNECs compared to the mock control, peaking at 4 dpi (Fig. 7B). However, compared to VR740, IP-10 and IFN-λ1 secretion were lower on 6 and 4 dpi, respectively, following 22050721 infection. As in the case of HCoV-NL63, very little secretion of IFN-λ2/3 and IFN-β was observed in HNECs in response to HCoV-229e infection. In BCi, infection with HCoV-229e strongly induced IP-10 secretion peaking on 4 dpi for 2205298 and 6 dpi for VR740 (Fig. 7C). Modest increases in IFN-λ2/3 and IFN-β secretion were also observed with a similar peak for 2205298 on 4 dpi and VR740 on 6 dpi (Fig. 7C). In AT2 cells, modest increases in the secretion of all four cytokines were observed on 4 dpi and/or 6 dpi (Fig. 7D). However, no difference between cytokine secretion was observed between strains. Together, these data indicate that infection with contemporary sHCoVs induces a similar cytokine profile following infection, but that kinetics and concentration can differ.

## DISCUSSION

Here, we demonstrated the utility of using specialized airway culture models to isolate novel contemporary sHCoV isolates from human nasal swabs. Using these models, we isolated 15 CoVs (71% success rate), which we further characterized for sequence variation, replication kinetics, and host response compared to the commonly used lab-adapted reference strains. Previous work by Dijkman et al. demonstrated the usefulness of air-liquid interface airway cultures for isolating sHCoVs. They recovered nine viruses using primary bronchial epithelial cells, only one of which was HCoV-NL63 (11). This mirrors our observation that HCoV-NL63 isolates are poorly recoverable in BCi cells. Komabayashi et al. recovered 29 CoVs from 36 nasal swab specimens in human bronchial epithelial cells and showed that HCoV-OC43 and HCoV-NL63 infected cultures produced infectious virus for at least 100 days (31). We did not measure viability or propagate the virus past 7 dpi. However, we did note that HCoV-229e caused obvious CPE in AT2 cells (data not shown) while no obvious CPE was observed with any other virus or cell type. In our study, we did not have any samples that were PCR+ for HCoV-HKU1, so we cannot comment on whether we could have recovered this virus using HNECs, BCi, or AT2 cells.

We observed that HCoV-229e isolates could be recovered or replicated in HNECs, BCi, and AT2 cells, similar to the lab-adapted HCoV-229e strain. HCoV-OC43 isolates were isolated in BCi, which was in direct contrast to the lab-adapted strain that failed to replicate in these cells. HCoV-NL63 isolates could only be recovered and replicated in HNECs, whereas the lab-adapted strain could replicate in all airway cell types. Importantly, while HNECs require a nasal brush specimen from a healthy adult and generation of stem cell-derived AT2 cells can be challenging, our results suggest that the immortalized BCi-NS1.1 cell line differentiated at an ALI could be used to reliably recover HCoV-229e and HCoV-OC43 isolates. Recovery of HCoV-NL63 in HNECs is consistent with a higher expression of ACE2 in HNECs compared to BCi and AT2 cells (12, 14). This suggests that the tropism of the lab-adapted CoVs in the airways is distinct from that of clinical CoV isolates. Even for HCoV-229e, which showed similar tropism between lab-adapted and contemporary CoV isolates, direct comparison revealed a slight difference in kinetics of viral replication and cytokine responses, indicating the importance of incorporating contemporary sHCoVs in future research.

While the ability to reliably recover CoV isolates is important, air-liquid interface cultures and stem-cell-derived cultures are difficult and costly to scale up to produce virus stocks. Here, we developed novel techniques to amplify and titrate virus stocks in immortalized cell lines for HCoV-229e using Huh7 cells and HCoV-NL63 using LLC-AT cells. While Huh7 cells have been widely used to propagate lab-adapted 229E stocks (26–28), we have now shown their additional usefulness for propagating clinical isolates. Importantly, we confirmed that there are very few amino acid changes observed for both HCoV-229e and HCoV-NL63 after two passages in these cells, indicating that virus stocks prepared in these cells can be used to reliably scale virus stocks for research (at least following 1–2 passages). Komabayashi et al. had also observed that HCoV-NL63 isolates could be propagated in LLC-MK2 cells. However, we noted that while contemporary HCoV-NL63 could also grow in LLC-MK2 cells in our hands, overexpression of ACE2 and TMPRSS2 increased viral titers and shortened the time to reach >80% CPE (4 days in LLC-AT vs >8 days for LLC-MK2), which was critical for measuring virus titers by infectivity assay. Therefore, LLC-AT cells are an improved cell line for studying clinical isolates of NL63.

Unfortunately, despite several attempts, we could not develop an immortalized cell culture system to amplify and titrate contemporary HCoV-OC43 isolates, though the lab-adapted strain replicates efficiently and causes CPE in many cell types. Other groups have also noted the difficulty of propagating HCoV-OC43 clinical isolates in immortalized cell lines (32). We attempted to increase the amount of clinical HCoV-OC43 isolates collected from the BCi cultures by pooling multiple wells, sampling >twice a day, and pooling samples collected on multiple days. While this produced larger “stocks,” we observed lower concentrations of virus genomes. Overcoming this bottleneck will be difficult without an alternative cell culture system. Contemporary HCoV-OC43 isolates showed a clear difference in tropism within the respiratory tract compared to the lab-adapted reference HCoV-OC43 strain. Whether this is related to differences in receptor usage or binding affinity, entry co-factor expression, or replication machinery is unclear. Based on our data, we cannot conclude that lab-adapted HCoV-OC43 does not replicate at all in human bronchial epithelial cells, given that BCi are derived from a single donor and there may be donor-to-donor variations in receptor expression. However, other groups have also noted that human bronchial epithelial cells show little to no replication of lab-adapted HCoV-OC43 (32, 33). Our data suggest that studies investigating HCoV-OC43 receptor usage should consider this discrepancy among isolates and donors. A cell line to propagate these viruses would also overcome the bottleneck of working with HCoV-OC43 isolates.

Of the three sHCoVs, HCoV-NL63 showed the least genetic variability compared to the lab-adapted strain. The exclusivity of contemporary HCoV-NL63 for replication in the nasal epithelium may shield HCoV-NL63 from the selective pressure of antibodies, in contrast to both HCoV-OC43 and HCoV-229e contemporary isolates that can replicate beyond the upper respiratory tract. Overall, the greatest variability in the sequence of the three sHCoVs compared to the lab-adapted reference strains was within the S gene. This is consistent with previous reports (8–10). Whether the mutations we observed in ORF1ab and S following passage in LLC-AT cells are also associated with enhanced replication in cell lines requires further analysis.

Sequence analysis showed that most amino acid changes within our HCoV-229e isolates compared to the reference strain occurred within the RBD, particularly within the RBLs responsible for hAPN binding. Previous genetic and serological studies suggest substantial antigenic evolution has occurred within the HCoV-229e RBL over the last 60 years (8, 10, 34). Wong et al. demonstrated that HCoV-229e RBDs sampled between 1967 and 2015 could be classified into six classes based on RBL sequence (24). Interestingly, the RBD sequence of our isolates differed from RBDs categorized in these classes, suggesting further genetic evolution into a new RBD class. While Wong et al. found that the more recent RBD classes (V and VI) bound hAPN with a higher affinity than classes I–IV, the affinity of more recent HCoV-229e RBDs (post-2015) requires further investigation. In contrast to HCoV-229e, HCoV-NL63 isolates demonstrated minimal variation in the RBD compared to the reference strain. Instead, the Unique domain of HCoV-NL63 showed the most variation in S1 compared to Am-1. While this domain is not required for binding to hACE2 (35), it has been speculated to bind to heparin sulfate as an attachment factor (36).

Recombination is an important mechanism of genetic variation in CoVs that can extend the host range. Intraspecies recombination has been documented to occur in HCoV-229e, HCoV-NL63, and HCoV-OC43 sequences (10, 21, 22, 37). We identified one in three HCoV-229e isolates and one in eight HCoV-OC43 isolates demonstrated evidence of genetic recombination. Two studies found that HCoV-229e recombinants were rare, although several sequences were identified with breakpoints between S and M or N genes (38). Interestingly, recombination with breakpoints in ORF1ab and S has been demonstrated between HCoV-229e, bat HCoV-229e-related CoVs, and alpaca HCoV-229e-related CoVs (39). Recombination analysis of previous HCoV-OC43 sequences by Pollett et al. demonstrated S and N both had recombination breakpoints at similar positions to those of 17101604 (40).

Overexpression of TMPRSS2 increased the ability of all HCoV-229e isolates to replicate in Huh7 cells. For the contemporary isolates, overexpression of TMPRSS2 also meant that their replication was much more like that of the lab-adapted strain. A study by Shirato et al. showed that two HCoV-229e clinical isolates, one from 2004 and one from 2008, were more dependent on TMPRSS2 for entry compared to the lab strain. They also showed that passage of a HCoV-229e clinical isolate 20 times through HeLa cells induced several mutations within the S gene that reduced TMPRSS2 usage and reduced replication fitness in human bronchial epithelial cells (41). A similar increase in replication was observed for the HCoV-NL63 isolates in LLC-MK2 cells overexpressing ACE2 and TMPRSS2. Others have shown that the early stages of HCoV-NL63 entry into human airway epithelial cells, but not LLC-MK2 cells, are mediated by TMPRSS2 (17). Therefore, it’s unclear whether TMPRSS2 is contributing to the increased replication observed in LLC-AT cells or whether this increase is attributable to increased ACE2 expression.

There are some limitations to our study. Firstly, we did not attempt to recover virus from all nasopharyngeal swabs using all three respiratory models because we were constrained by the volume of the sample. For example, we cannot definitively say that HCoV-OC43 and HCoV-229e isolates can be recovered in HNECs since we only attempted to recover HCoV-NL63 using these cells. However, given that we have shown HCoV-229e isolates replicate well in HNECs, we speculate that they could be recovered in HNECs. In addition, we did not attempt to recover all HCoV-OC43 isolates in AT2 cells. Thus, while we failed to recover two, it is unclear whether these cells would be refractory to infection with all HCoV-OC43 isolates. Our sample size for virus isolation experiments in primary HNECs was small (*n* = 2 donors), and therefore, we cannot speculate whether donor-dependent variation in the ability to recover isolates or replication efficiency of contemporary sHCoV isolates would occur. Finally, our experiments examining replication kinetics and cytokine expression in the respiratory tract only compared one contemporary isolate each to the lab-adapted reference strain. Given the genetic diversity between the isolates, particularly for HCoV-229e, it is possible that the isolate selected for analysis may not reflect all circulating HCoV-229e isolates.

Overall, here we have shown that contemporary sHCoVs differ from lab-adapted reference strains and should be used for the study of virus biology and evaluation of medical countermeasures. Furthermore, the technical advances described in our study should make working with these HCoVs easier and facilitate future research endeavors.

## MATERIALS AND METHODS

### Cell lines

MRC-5 cells (CCL-21, ATCC) were maintained in DMEM supplemented with 50 U/mL penicillin, 50 µg/mL streptomycin, 1 mM sodium pyruvate, 20 mM HEPES, 0.1 mM non-essential amino acids, 2 mM Glutamax, 0.18% (vol/vol) sodium bicarbonate, and 10% (vol/vol) FBS. Huh7 cells were maintained in DMEM supplemented with 50 U/mL penicillin, 50 µg/mL streptomycin, 2 mM Glutamax, and 10% (vol/vol) FBS. LLC-MK2 cells were maintained in MEM supplemented with 50 U/mL penicillin, 50 µg/mL streptomycin, 15 mM HEPES, 2 mM Glutamax, and 5% (vol/vol) FBS. Basal-like human airway progenitor cells (BCi-NS1.1 [42]) were maintained in Pneumacult-Ex Plus medium (StemCell Technologies, Cat. 05040) supplemented with 96 ng/mL hydrocortisone (StemCell Technologies, Cat. 07925) and 50 U/mL penicillin and 50 µg/mL streptomycin.

### Genetically modified cell lines

LLC-AT cells were generated by transducing LLC-MK2 cells with lentiviral constructs expressing human ACE2 (pHAGE2 containing the angiotensin-converting enzyme 2 gene; NR-52512, BEI Resources, NIAID, NIH) and TMPRSS2 (pscALPSblasti-TMPRSS2 Blasti, Addgene plasmid #158088, a gift from Jeremy Luban). LLC-AT cells were maintained in MEM supplemented with 50 U/mL penicillin, 50 µg/mL streptomycin, 15 mM HEPES, 2 mM Glutamax, and 5% (vol/vol) FBS. Huh7 overexpressing TMPRSS2 (Huh7-T2) were made by transducing Huh7 cells with a lentiviral construct expressing TMPRSS2 (pscALPSblasti-TMPRSS2 Blasti, Addgene plasmid, Cat. 158088, a gift from Jeremy Luban). Transduced cells were selected by supplementation of culture media with 2 µg/mL Blasticidin (Gibco, Cat. A1113903) until negative control cells were dead. TMPRSS2 expression was confirmed by surface staining selected cells with 5 µg/mL goat anti-TMPRSS2 PE (BioLegend, Cat. 378403) antibodies and analyzed by flow cytometry. Huh7-T2 cells were maintained in DMEM supplemented with 50 U/mL penicillin, 50 µg/mL streptomycin, 2 mM Glutamax, 10% (vol/vol) FBS, and 1 µg/mL Blasticidin.

### Nasal brushing for nasal epithelial cells

Healthy adults were recruited for this study under ethics approval HREC/35132. Nasal epithelial cells were sampled and cultured in Pneumacult-Ex Plus medium (StemCell Technologies, Cat. 05040) supplemented with 96 ng/mL hydrocortisone (StemCell Technologies, Cat. 07925), as well as 50 U/mL penicillin, 50 µg/mL streptomycin, and 0.25 µg/mL amphotericin B (ThermoFisher Scientific, Cat. 15290018), as described previously (12, 43, 44).

### Differentiation of airway epithelial cells

Nasal epithelial cells or BCi-NS1.1 cells were seeded at 300,000 cells/well in 12 mm transwell membrane supports (Corning, Cat. 3460) or 150,000 cells/well in 6.5 mm transwell membrane supports (Corning, Cat. 3450) and cultured in Pneumacult-Ex Plus medium in apical and basolateral chambers. Upon reaching confluence (2–3 days post-seeding), apical medium was removed to expose cells to ambient air; medium in the basolateral chamber was replaced with Pneumacult ALI maintenance medium (Stemcell Technologies, Cat. 05001) supplemented with 4 µg/mL heparin (Stemcell Technologies, Cat. 07980), 480 ng/mL hydrocortisone, 50 U/mL penicillin, and 50 µg/mL streptomycin. HNEC cultures were also supplemented with 0.25 µg/mL amphotericin B. Medium in the basolateral chamber was replaced with fresh Pneumacult ALI maintenance medium every 2 to 3 days. The apical surfaces of cultures were washed with PBS (+Ca^2+^/Mg^2+^) once a week to remove mucus accumulation. BCi cells were cultured for a minimum of 28 days to form differentiated, polarized human bronchial epithelial cell cultures, while nasal epithelial cells were cultured at an ALI for 42 days to form differentiated nasal epithelial cell (HNEC) cultures. Amphotericin B was removed from basolateral medium at 28 days post-airlift.

### Generation of hESC-derived AT2 cells

Human embryonic H9 (female) stem cells were differentiated into lung AT2 cells as previously described (12, 13). Briefly, H9 stem cells were differentiated as a three-dimensional organoid for 18 days in flasks coated with Matrigel (Corning, Cat. 354230). Lung organoids were maintained for experiments between passages 2–8 and dissociated with TrypLE (Thermo Fisher Scientific, Cat. 12604013) and seeded onto Geltrex-coated plates (Gibco, Cat. A1413201). Cells were then maintained for a further 7–10 days in two-dimensional culture until infection at >70% confluency.

### Lab virus stocks

HCoV-NL63 Lab virus (Amsterdam-1) was a kind gift from Prof Lia van der Hoek, University of Amsterdam, shared by Prof Kirsten Spann, Queensland University of Technology, with permission. Viral stocks were prepared from supernatants of infected LLC-MK2 cells. HCoV-229e (VR740 [Inf1]) and HCoV-OC43 (VR1558) were kind gifts from Prof. Nathan Bartlett, University of Newcastle. Viral stocks were prepared from supernatants of infected MRC-5 cells.

### Clinical material

Stored nasopharyngeal specimens were shared by the Victorian Infectious Disease Reference Laboratory, Melbourne, Australia, for the isolation of contemporary HCoVs as approved by the University of Melbourne Human Ethics Committee (reference 2024-31337-61425-3). Briefly, samples were collected by referring to pathology services and stored at –80°C. Samples were typed using the following primers CVHCoV-229e-F 5′ TCACATGTTGTACGGCTAGTGATAAA 3′, CVHCoV-229e-R 5′ ACCCACCATTTGAATAAACAACCT 3′, probe CVHCoV-229e 5′ VIC-AGCAAGCTCATTACTAAGTCTA-MGBNFQ 3′, HCor-F 5′ AAATTTTATGGTGGCTGGAATAATATGTT 3′, HCoronaRT-Ra 5′ TAGGCATAGCTCTRTCACAYTT 3′, HCoronaRT-Rb 5′ TTGGCATRGCACGATCACAYTT 3′ and probe Corona 5′ FAM-TGGGTTGGGATTATC-MGBNFQ 3′ which can detect the presence of HCoV-229e or other non-HCoV-229e, non-HCoV-HKU1 CoVs (either HCoV-NL63 or HCoV-OC43). No samples containing HCoV-HKU1 were analyzed. A total of 21 samples were used to recover infectious HCoVs: 5 HCoV-229e and 16 other HCoVs. Two samples were lost due to fungal contamination. A list of the samples and their collection dates is shown in Table 2.

### Isolation of infectious virus from clinical specimens

200 µL of clinical specimen was diluted with 600 µL of PBS and centrifuged at 2,000 rpm for 5 min. Clarified sample (200 µL) was added to each well of either airway epithelial cells or AT2 cells and incubated for 2 h at 33°C. Following virus adsorption, the virus inoculum was removed, and cells were incubated at 33°C for up to 7 days. AT2 cells were cultured in 500 uL of media. Supernatant was collected daily, and 500 µL of media was re-added to the cells. For airway epithelial cells, 200 µL of PBS was added to the apical surface, incubated for 10 min at 33°C, and collected. All viruses were stored at −80°C.

### Next-generation sequencing

Stocks of HCoV-NL63, HCoV-229e, and HCoV-OC43 were extracted using the QIAamp Viral RNA Mini Kit. Complementary DNA (cDNA) was synthesized from 15 µL of each sample using random hexamers and the ProtoScript II first strand cDNA synthesis kit (New England Biolabs, Ipswich, MA, USA), followed by the NEBNext Ultra II Non-Directional RNA Second Strand Synthesis Module (New England Biolabs). Indexed libraries were prepared using the Twist Total Nucleic Acids Library Preparation Kit for Viral Pathogen Detection and Characterization (Twist Biosciences, South San Francisco, CA, USA) following the manufacturer’s protocol. Pre-capture libraries were quantified using QubitTM dsDNA HS (Thermo Fisher Scientific) and qualified using Agilent Tapestation DNA HS reagents (Agilent Technologies, Santa Clara, CA, USA). Indexed libraries were pooled by mass prior to capture. Twist hybridizations followed the Twist Target Enrichment Standard Hybridization v1 Protocol using the Twist Comprehensive Viral Research Panel, according to the manufacturer’s instructions and with a 16 h incubation at 70°C. Libraries were quantified and qualified as above, before sequencing using a paired-end 150 bp chemistry with a P1 cartridge on an Illumina NextSeq2000 (Illumina, San Diego, CA, USA).

### Sequence analysis

Reads were mapped to viral reference genomes for relevant taxa accessed from NCBI Virus (https://www.ncbi.nlm.nih.gov/labs/virus/vssi/#/) iteratively using Minimap2 to generate consensus sequences (45). To generate sequence similarity plots (Simplots), full genome nucleotide sequence alignments were input into Simplot++ for Simplot analysis using the Jukes-Cantor substitution model with a window size of 100 bp and a step size of 20 bp (46).

### Phylogenetic analysis

Sequences were aligned using MAFFT version 7.526 (47). Time-resolved trees were constructed using the maximum likelihood method with generalized-time reversible (GTR) nucleotide substitution model as implemented in IQ-TREE2 version 2.3.3 (48) and the least-square dating method (49). Trees were visualized using ggtree version 3.10.1 (50), and sequences were grouped by lineage or genotype as described by Ye et al. (21).

### Recombination analysis

Sequence alignments generated using MAFFT version 7.526 (47) were analyzed using the recombination detection program version 5 (RDP5) (51). Similarity plots were generated using the RDP method and a 30 bp window size.

### Production of HCoV-229e and HCoV-NL63 viral stocks in immortalized cell lines

Airway epithelial cell supernatants (HNEC-derived HCoV-NL63 and BCi-derived HCoV-229e) were diluted with MEM (1:10) and inoculated onto confluent flasks of either LLC-AT or Huh7 cells for 1 h at 33°C. Following adsorption, additional media (1% FBS and 1 µg/mL TPCK-trypsin for Huh7 and 2% FBS and 1 µg/mL TPCK-trypsin for LLC-AT) were added. Flasks were cultured until approximately 80%–100% CPE was observed (days 3–4 for HCoV-229e and days 6–7 for HCoV-NL63). Supernatants were clarified by centrifugation at 4,000 rpm for 10 min, and virus stock aliquots were stored at −80°C. Stock titers were determined by virus titration as described below.

### Replication kinetics in immortalized cell lines

Confluent monolayers of Huh7, Huh7-T2, LLC-MK2, and LLC-AT cells seeded into 24-well plates were washed with 500 µL plain MEM before inoculation with 100 µL of either HCoV-229e diluted to a multiplicity of infection (MOI) of 0.001 or HCoV-NL63 diluted to an MOI of 0.01 for 1 h at 33°C. Following removal of the virus inoculum, the cells were washed three times and cultured at 33°C up to 4 dpi. Supernatants were collected daily for virus titration and qRT-PCR as described below.

### Virus infection of human airway epithelial cells

Prior to infection, airway cultures were washed once with pre-warmed PBS (+Ca^2+^/Mg^2+^) to remove mucus on the apical surface. Cells were inoculated with 100 µL HCoV-229e diluted to an MOI of 0.01, HCoV-NL63 diluted to 10^7^ genome copies, or HCoV-OC43 diluted to an MOI of 0.1 for 2 h at 35°C. The inoculum was removed, and the apical surface was washed three times with PBS, and the third wash was collected as the day 0 sample. On days 1–7 post-infection, 200 µL PBS was added to the apical chamber and incubated at 35°C for 10 min before samples were collected and stored at −80°C. Basolateral medium was replaced every second day with ALI maintenance medium. Apical samples were assayed by virus titration as described above and qPCR (E gene copies) as described below.

### Virus infections of lung AT2 cells

For infection of lung AT2 cells, cells were inoculated with 10^4^ TCID_50_ of HCoV-229e, 10^7^ genome copies of HCoV-NL63, or 10^4^ TCID_50_ of HCoV-OC43 in 100 µL for 1 h at 35°C. Cells were washed three times with PBS, the third wash was collected as the day 0 sample, and cells were refed with lung AT2 medium. Supernatant samples were collected every day for 7 days, and the media were replenished on days 1–6. Supernatant samples were assayed by virus titration and qRT-PCR as described below.

### Quantification of infectious virus titer

Virus titrations were performed in 96-well plates with confluent monolayers of LLC-AT, Huh7 cells, and Huh7-T2 cells. For Huh7 and Huh7-T2 cells, plain DMEM was used to wash cells and replaced with 180 µL media containing 1% FBS and 1 µg/mL TPCK-trypsin. For LLC-AT cells, plain MEM was used to wash cells and replaced with 180 µL media containing 2% FBS and 1 µg/mL TPCK-trypsin. Each sample was titrated in quadruplicate by adding 20 µL supernatant to the first well and performing 10-fold serial dilutions. Cells were incubated at 33°C and assessed microscopically for virus-induced CPE on day 3 for HCoV-229e or day 7 for HCoV-NL63. Virus titers are expressed as mean log_10_TCID_50_/mL.

### RNA extraction and qRT-PCR

RNA was extracted as per the manufacturer’s recommendation using either the QiaCube HT (Qiagen) and QiaAmp 96 Virus QiaCube HT kit (Qiagen, Cat. 57731) or QIAamp Viral RNA Mini Kit (Qiagen, Cat. 52904). qRT-PCR reaction was set up using SensiFast Probe No-ROX One-Step Kit (Bioline, Cat. BIO-76005) using the following primers/probes: HCoV-NL63 Forward: 5′-AGGTTGACTTGTATAATGGTGCT-3′, HCoV-NL63 Reverse: 5′-GCCAACACAAAGAAAAATATCA-3′ and HCoV-NL63 Probe: 5′- TGCCGAAGAGCCTGTTGTTGGT-3′; HCoV-229e Forward: 5′- ATGTGTACCACATTTACCAATCA-3′, HCoV-229e Reverse: 5′- TCCAAACTGAAGAATAACAATGA-3′ and HCoV-229e Probe: 5′- TGCACATAGACCCTTTCCCTAAACG-3′; HCoV-OC43 Forward: 5′-TGTTTATGGCTGATGCTTATCT-3′, HCoV-OC43 Reverse: 5′- AAGGTATTACACATACCGCAAA-3′ and HCoV-OC43 Probe: 5′- CACTGTGTGGTATGTGGGGCAAAT-3′. Serial 10-fold dilutions of plasmid encoding the viral sequence target of interest were used to generate a standard curve and calculate the virus genome copies in the samples.

### Cytokine bead array

Cytokine/chemokine concentrations in supernatants or basolateral medium were analyzed using the LEGENDplex human anti-virus response panel (Biolegend, Cat. 740390) following the manufacturer’s instructions. Samples were run on a BD FACSCanto II and analyzed using LEGENDplex Data Analysis Software Suite.

### Immunofluorescence

Airway epithelial cultures in transwells were fixed in 10% formalin for 30 min at room temperature before three PBS washes were performed. Samples were blocked with 5% normal goat serum (Thermo Fisher Scientific, Cat. 31873) containing 0.1% Triton X-100 (Sigma Aldrich, Cat. X100-100ML) in PBS for 1 h at room temperature. Samples were stained with primary antibodies (human APN, *In Vitro* Technologies, Cat. NBP2-77451, Dilution 1:50) overnight at 4°C. Samples were washed three times in PBS and incubated with Goat anti-Rabbit IgG (H+L) AF568 (Cat. 11011, 1:500) at room temperature for 1 h. Slides were washed three times with PBS and counterstained with 4′,6-diamidino-2-phenylindole, dihydrochloride (DAPI, Thermo Fisher Scientific, Cat. D1306) for 5 min at room temperature in the dark. Membranes were cut from transwells and mounted onto Menzel Gläser, SuperFrost Plus glass slides (Rowe Scientific PTY LTD, Cat. GM2920) using VectaShield PLUS Mounting Medium (Vector Labs, Cat. VEH190010). Slides were imaged on a Zeiss LSM980 confocal microscope and analyzed with Zen software.

## Data Availability

Virus isolates are available upon request. All virus sequences have been deposited into GenBank (accession numbers: PV827139–PV827152).

## SUPPLEMENTAL MATERIAL

Supplemental figures jvi.00684-25-s0001.pdf Fig. S1 to S3.

ASM does not own the copyrights to Supplemental Material that may be linked to, or accessed through, an article. The authors have granted ASM a non-exclusive, world-wide license to publish the Supplemental Material files. Please contact the corresponding author directly for reuse.
