# Supplementary material for: Contemporary seasonal human coronaviruses display differences in cellular tropism compared to laboratory-adapted reference strains
Source: J Virol. 2025 Aug 27;99(9):e00684-25. doi: 10.1128/jvi.00684-25 (PMC12456146; doi:10.1128/jvi.00684-25)

Supplementary Data

Sup. Figure 1. Isolation of contemporary sHCoVs. Representative maximum intensity z-projections (top) and XZ orthogonal projections (bottom) of confocal images of HNEC (A) and BCi (B) cultures immunolabelled against APN (red) with DAPI (blue) to indicate the nucleus. (C) Confocal microscopy images of Lung AT2 cultures immunolabelled against APN (red) and DAPI (blue). Scale bar, 20 µm. Log_{10} genome copies/mL of HCoV-NL63-18091206 (D) and HCoV-OC43 isolates in AT2 cells (E) demonstrating lack of virus recovery. Log_{10} genome copies/mL of lab-adapted HCoV-OC43 (VR-1558) in BCi (F) and AT2 cells (G) from 0 to 7 dpi. For (D), the mean from 4 pooled replicates is shown. For (E), (F) and (G) the mean ± SD from 4 un-pooled replicates is shown. (D) and (E) represents data generated from a single attempt to recover virus using a nasal swab specimen. (F) and (G) are representatives of two independent experiments each with 3 replicates.

Sup. Figure 2. Sequence analysis of sHCoVs. Analysis of HCoV-229e S1 (D), HCoV-NL63 S1 (E) and HCoV-OC43 S1 (F) from contemporary isolates compared to the reference strain. Protein domains are annotated in blue and receptor binding loops (RBLs) or sialic acid binding loops (SBL) are annotated in orange. Dots indicate the same amino acid as the reference sequence.

Sup. Figure 3. Recombination analysis of sHCoVs. Recombination pattern and breakpoints of A) HCoV-HCoV-229e/Australia/22050721/2022 with China/BIME365-75/2019 and Japan/Fukushima_H829/2020, and B) HCoV-HCoV-OC43/Australia/17101604/2017 with Kenya/KLF_01/2018 and Japan/Fukushima_H148/2018. The recombinant region is shown in red with the 95% and 99% breakpoint confidence intervals shown in dark and light grey, respectively.

Supplementary Figure 1 - Isolation of contemporary seasonal hCoVs

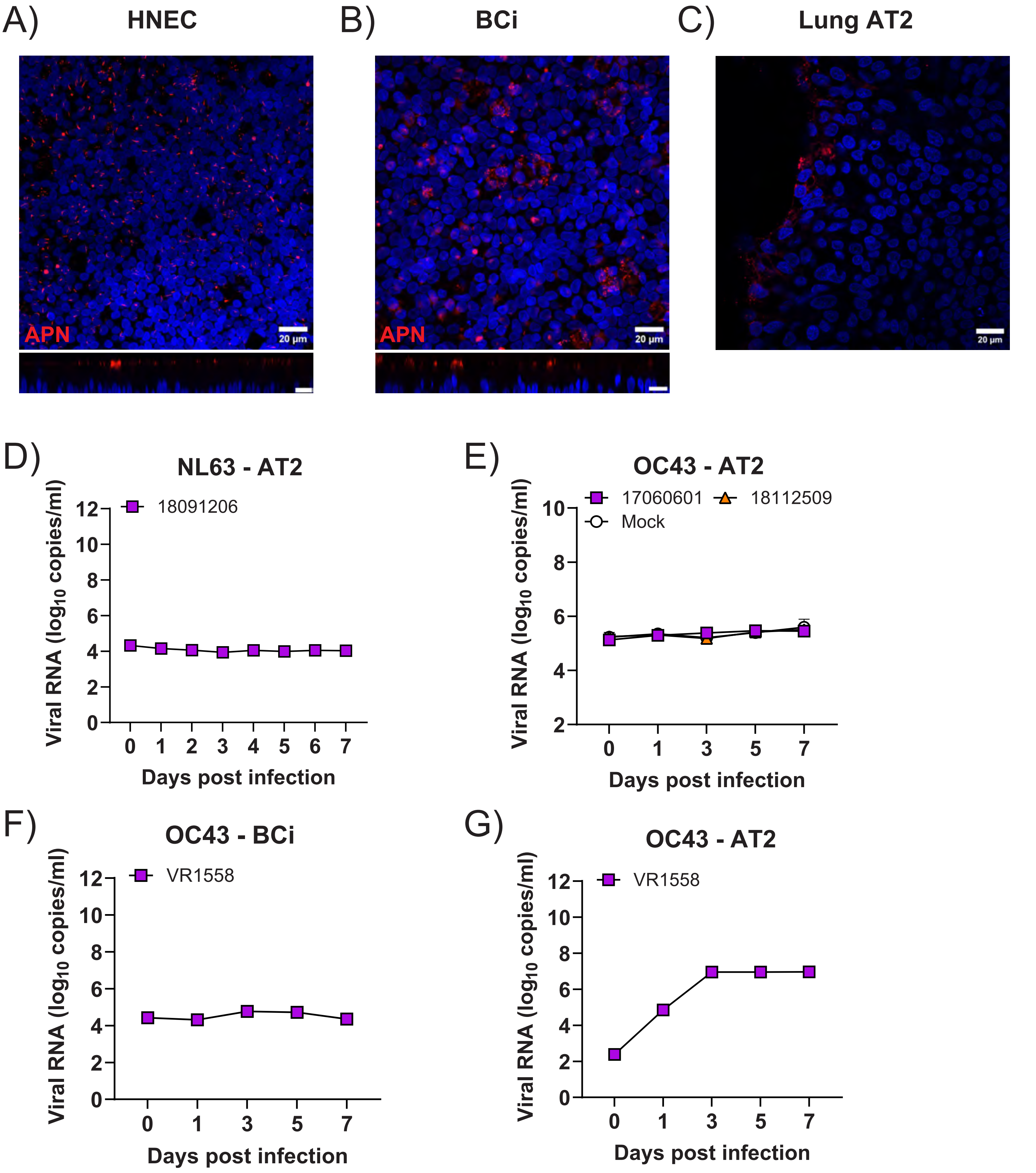

Supplementary Figure 2 - Alignment of S1 sequences

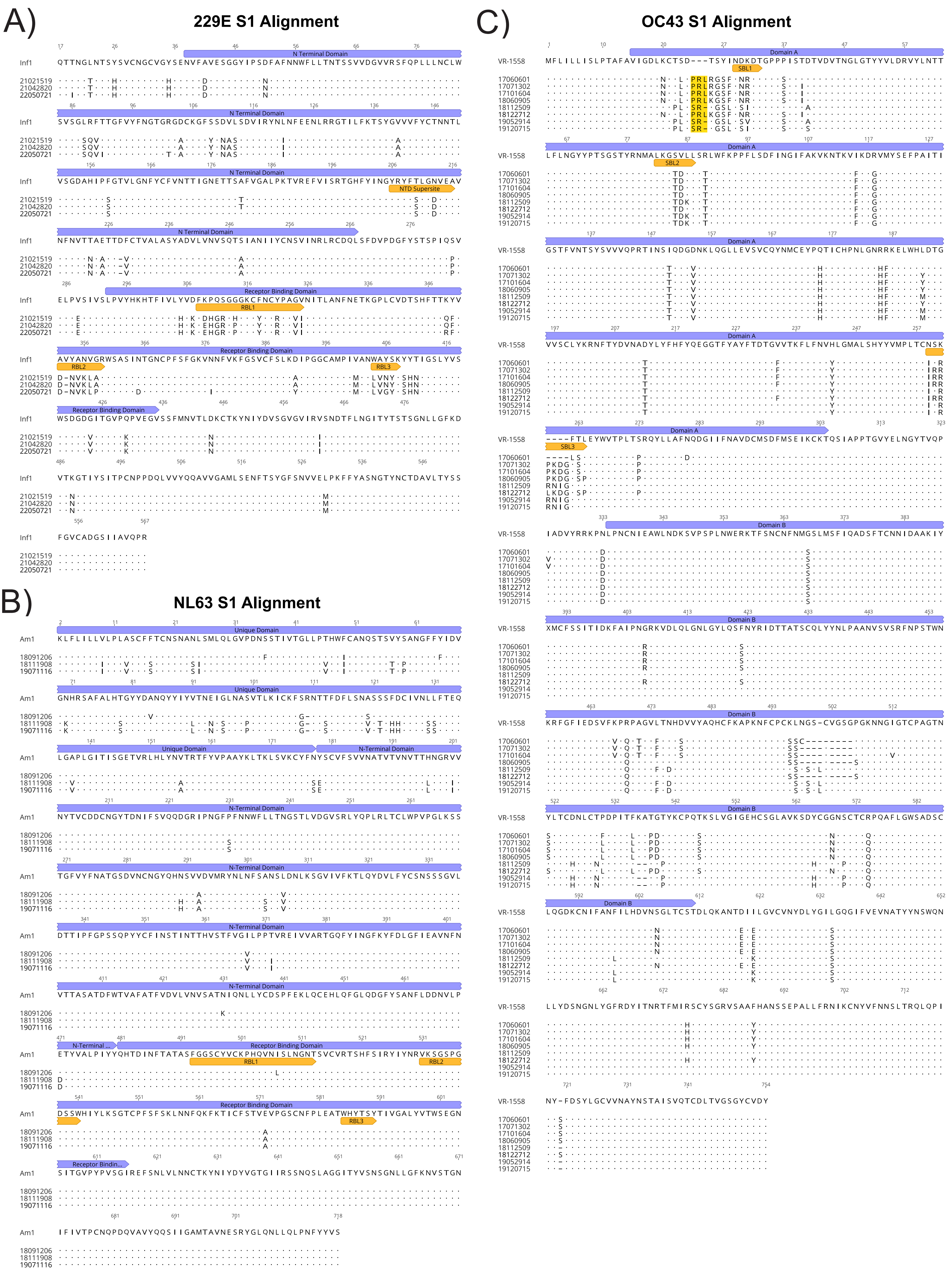

Supplementary Figure 3 - Recombination Analysis

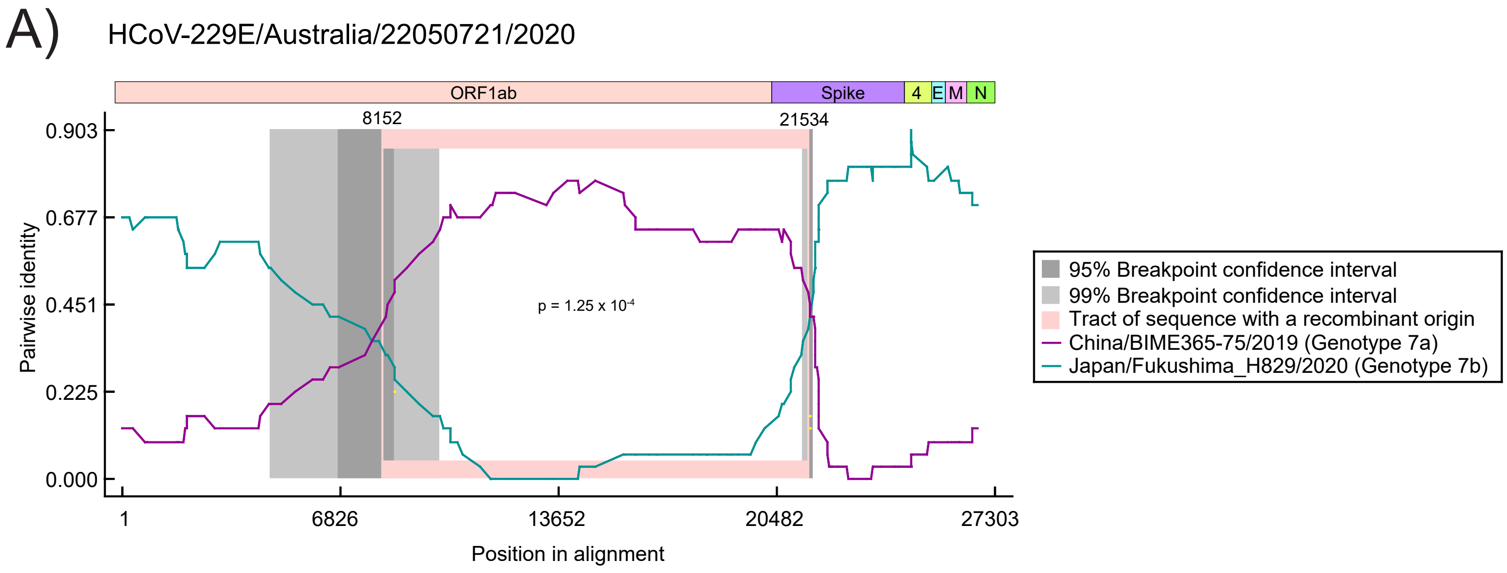

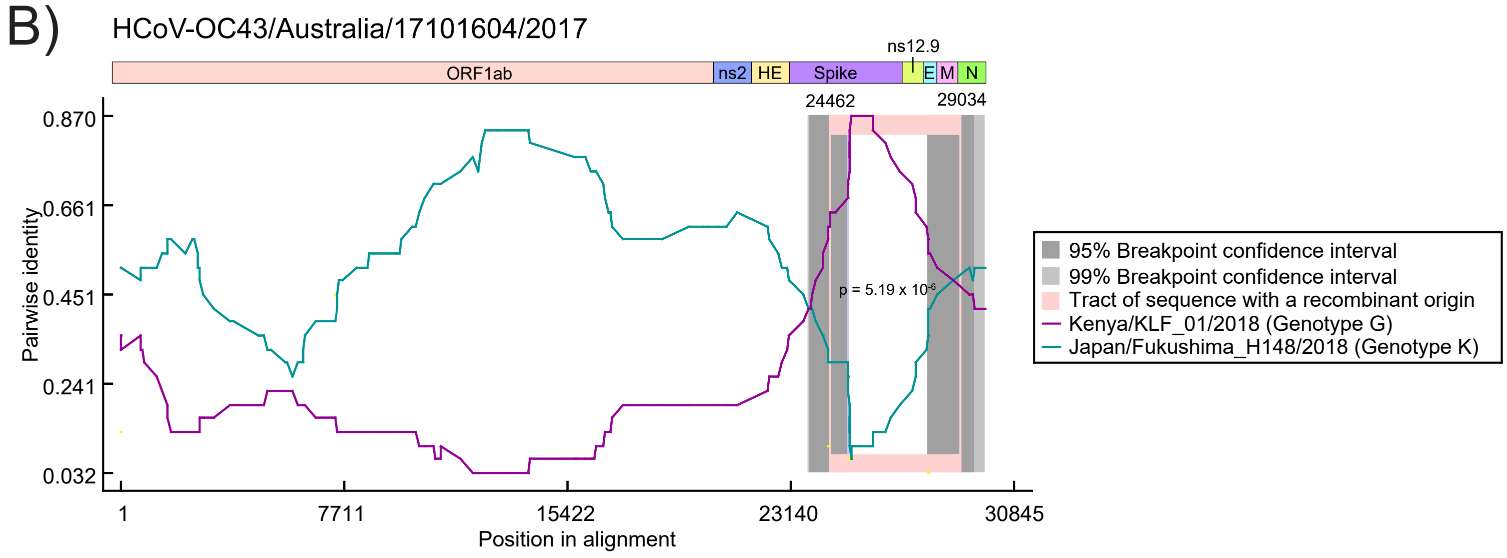

Supplement: Supplemental figures — Fig. S1 to S3. [file jvi.00684-25-s0001.pdf]
